# Crystal structure of seladelpar, C_21_H_23_F_3_O_5_S, from synchrotron powder diffraction data and density functional theory

**DOI:** 10.1107/S2056989026007875

**Published:** 2026-08-04

**Authors:** Jacob K. Salazar, James A. Kaduk, Anja Dosen, Megan Rost, Thomas N. Blanton

**Affiliations:** a North Central College, Department of Chemistry 131 S Loomis St Naperville IL 60540 USA; b North Central College, Department of Physics 131 S Loomis St Naperville IL 60540 USA; c Illinois Institute of Technology, Department of Chemistry, 3101 S. Dearborn St., Chicago IL 60616, USA; d ICDD, 12 Campus Blvd., Newtown Square PA 19073-3273, USA; University of Aberdeen, United Kingdom

**Keywords:** powder diffraction, seladelpar, Livdelzi, Rietveld refinement, density functional theory

## Abstract

The crystal structure of seladelpar, C_21_H_23_F_3_O_5_S [systematic name 2-(4-{(2*R*)-2-ethoxy-3-[4-(trifluoromethyl)phenoxy]propylsulfanyl}-2-methylphenoxy)acetic acid], has been solved and refined using synchrotron X-ray powder diffraction data, and optimized using density functional theory techniques. Seladelpar crystallizes in space group *P*2_1_2_1_2_1_ and the mol­ecule adopts a U-shaped conformation. The crystal structure is characterized by layers lying parallel to the *ab* plane. One classical O—H⋯O hydrogen bond links the carb­oxy­lic acid group and an ether O into chains propagating along the *b*-axis direction and the chains are consolidated by a weak C—H⋯S hydrogen bond.

## Abstract 2

The crystal structure of seladelpar has been solved and refined using synchrotron X-ray powder diffraction data, and optimized using density functional theory techniques.

## 1. Chemical context

Seladelpar, C_21_H_23_F_3_O_5_S (marketed as Livdelzi, as the lysine dihydrate salt), is used to treat primary biliary cholangitis, an autoimmune disease of the liver (Shukla & Misra, 2025 ▸). The systematic name (CAS Registry Number 851528-79-5) is 2-[4-[(2*R*)-2-eth­oxy-3-[4-(tri­fluoro­meth­yl)phen­oxy]prop­yl]sulfanyl-2-methyl­phen­oxy]acetic acid.
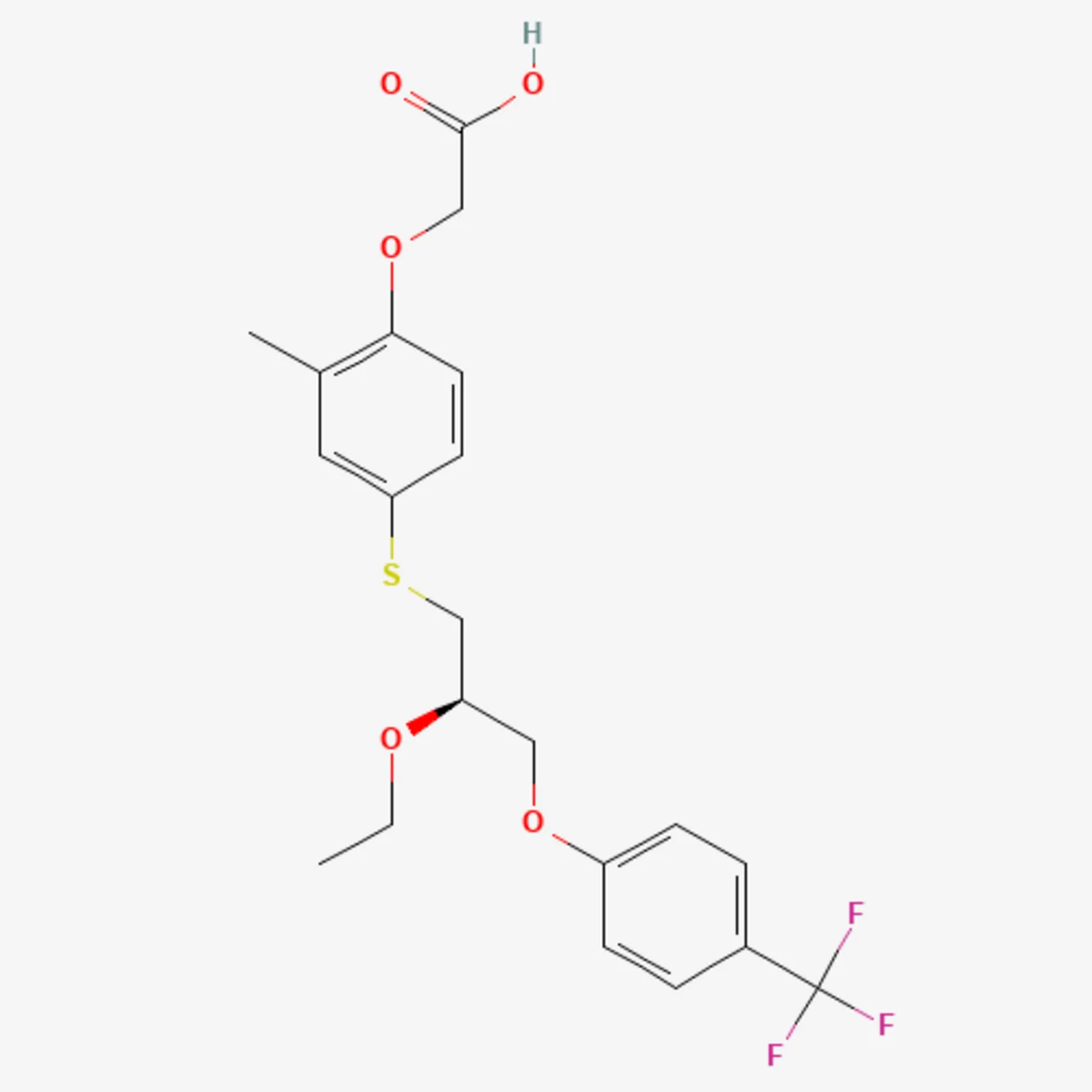


This work was carried out as part of a project (Kaduk *et al.*, 2014 ▸) to determine the crystal structures of large-volume commercial pharmaceuticals, and include high-quality powder diffraction data for them in the Powder Diffraction File (Kabekkodu *et al.*, 2024 ▸).

## 2. Structural commentary

**Figure 1:**
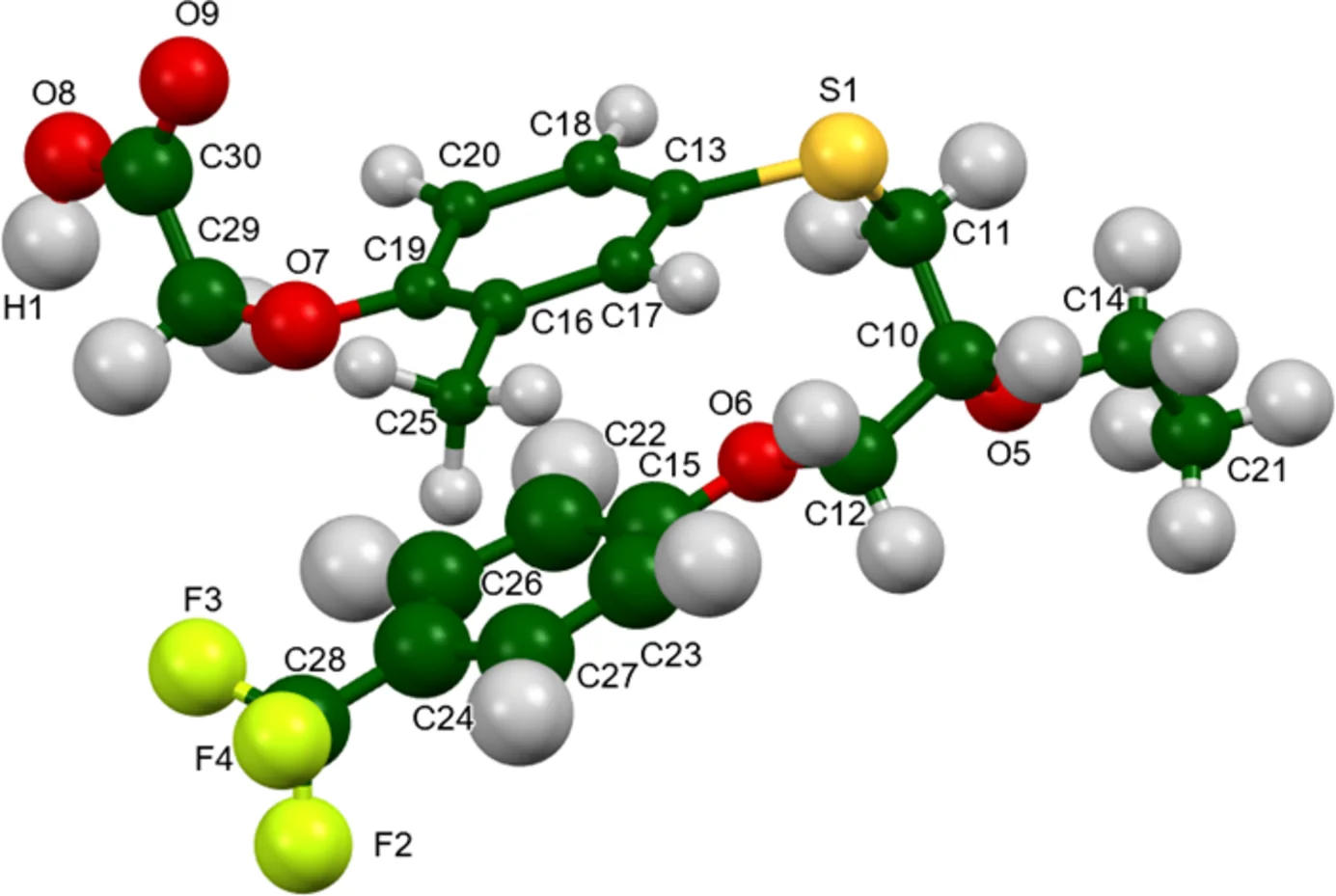
The mol­ecular structure of seladelpar, based on the Rietveld refinement, with the atom numbering. The atoms are represented by 50% probability spheroids.

**Figure 2:**
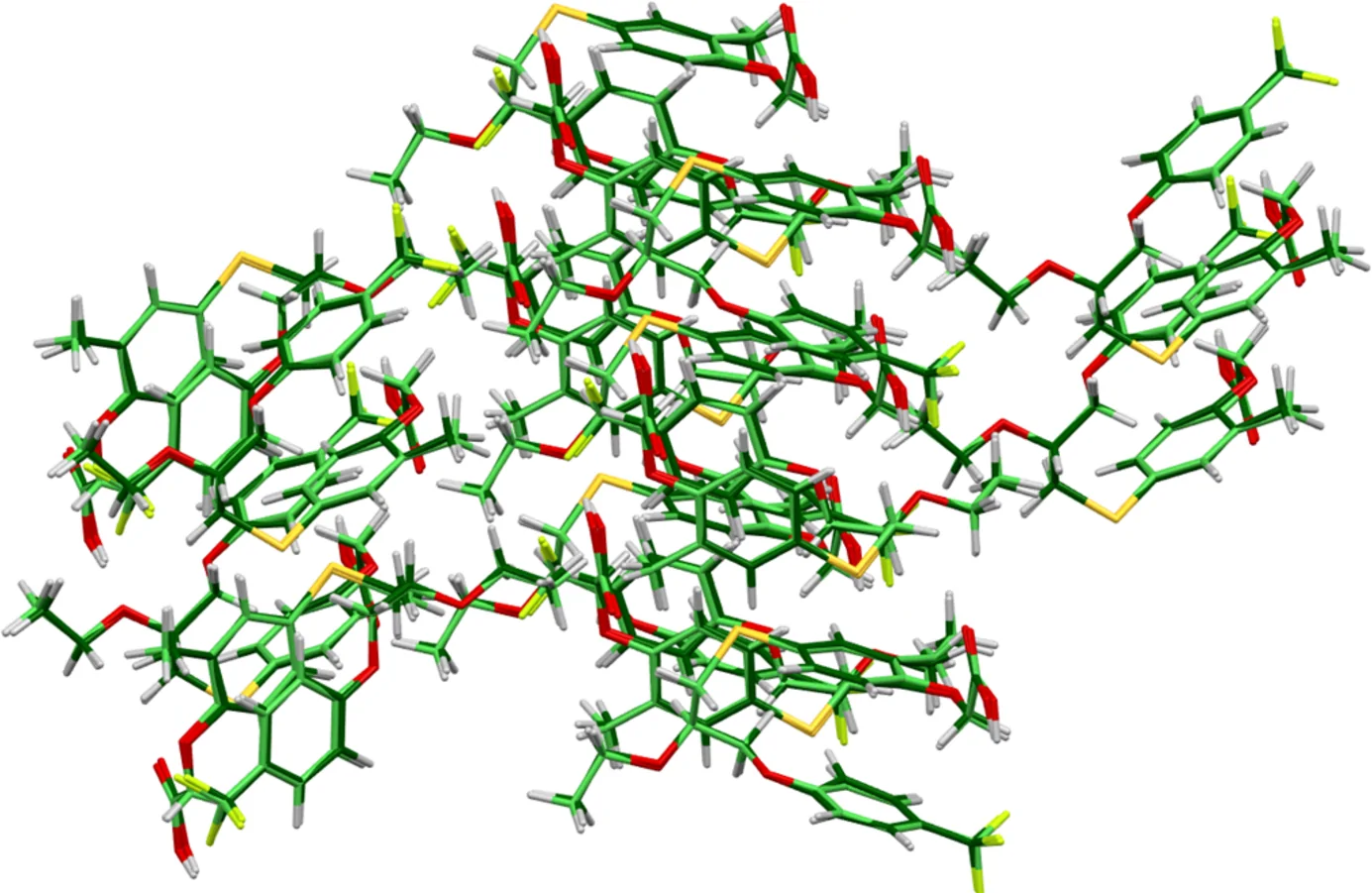
Comparison of the Rietveld-refined (colored by atom type) and *VASP*-optimized (pale green) structures of seladelpar, calculated using the *Mercury* CSD-Materials/Search/Crystal Packing Similarity tool. The root-mean-square Cartesian displacement is 0.139 Å.

**Figure 3:**
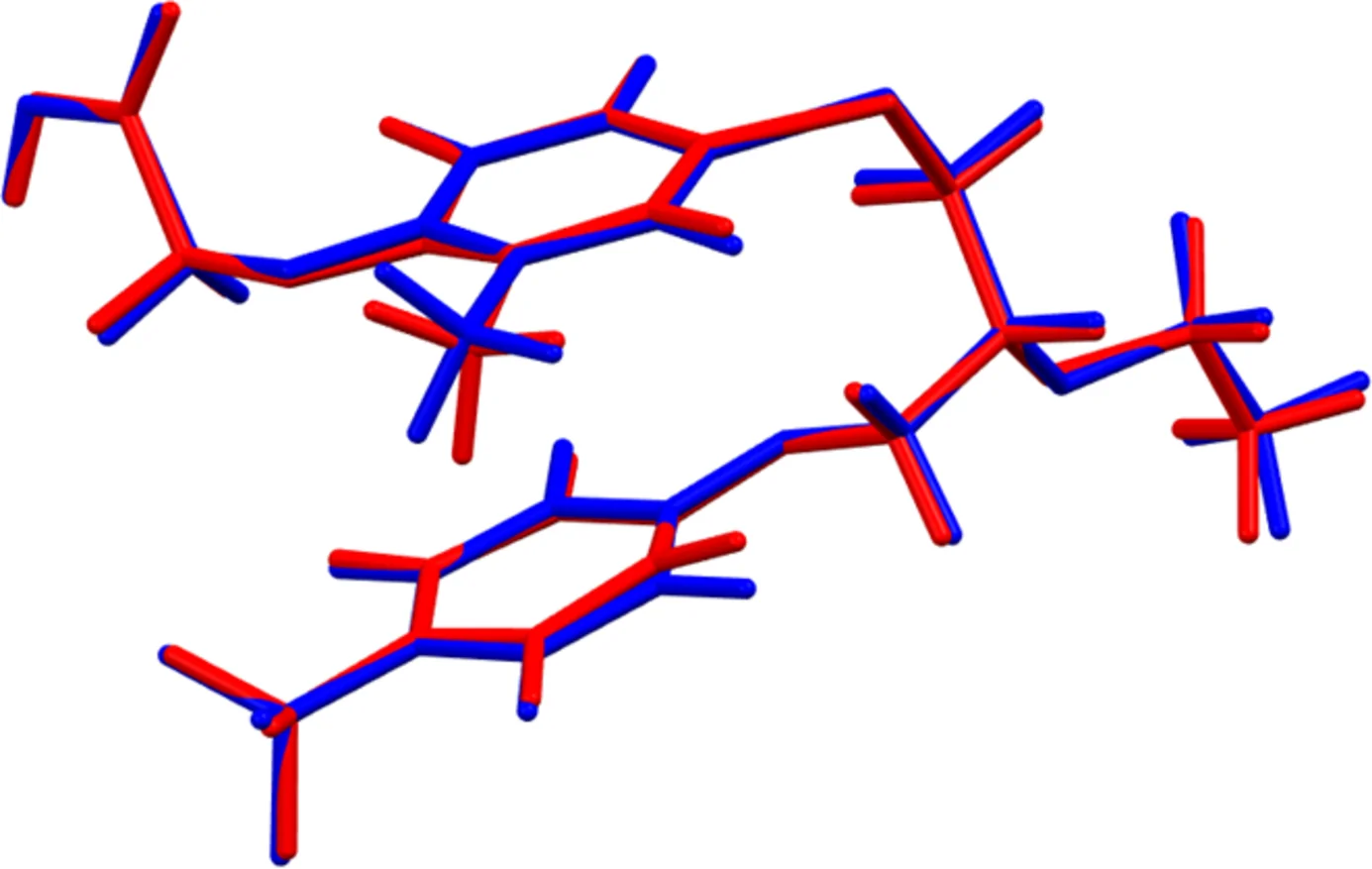
Comparison of the refined structure of seladelpar (red) to the *VASP*-optimized structure (blue). The comparison was generated using the *Mercury* Calculate/Mol­ecule Overlay tool; the r.m.s. difference is 0.112 Å.

The mol­ecular structure of seladelpar is illustrated in Fig. 1 ▸. The root-mean-square difference of the non-H atoms in the Rietveld-refined and *VASP*-optimized structures, calculated using the *Mercury* (Macrae *et al.*, 2020 ▸) CSD-Materials/search/crystal packing similarity tool is 0.139 Å (Fig. 2 ▸); the structures are essentially identical. The root-mean-square Cartesian displacement of the non-H atoms in the refined and optimized structures, calculated using the *Mercury* Calculate/mol­ecule overlay tool, is 0.112 Å (Fig. 3 ▸). The agreements are within the normal range for correct structures (van de Streek & Neumann, 2014 ▸). Given the normal representation of the mol­ecule in an extended conformation, the bent solid-state conformation with the phenyl rings approximately parallel may be unexpected. The remaining discussion will emphasize the *VASP*-optimized structure.

Almost all of the bond distances, bond angles, and torsion angles fall within the normal ranges indicated by a *Mercury* Mogul geometry check (Macrae *et al.*, 2020 ▸). Only the torsion angles involving rotation about the S1—C13 bond are flagged as unusual. They lie in broad valleys of bimodal 0/180° distributions of similar torsion angles, so the conformation of the mol­ecule is slightly unusual, but not unprecedented. The mean plane of the aromatic ring with the tri­fluoro­methyl substituent corresponds approximately with the (1
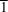
2) Miller plane, and the mean plane of the other phenyl ring is approximately (11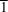). The bent shape of the mol­ecule is reflected in the normal S1—C11—C10—C12 (*gauche*) and O5—C10—C11—S1(*trans*) torsion angles (Table 1 ▸).

**Figure 4:**
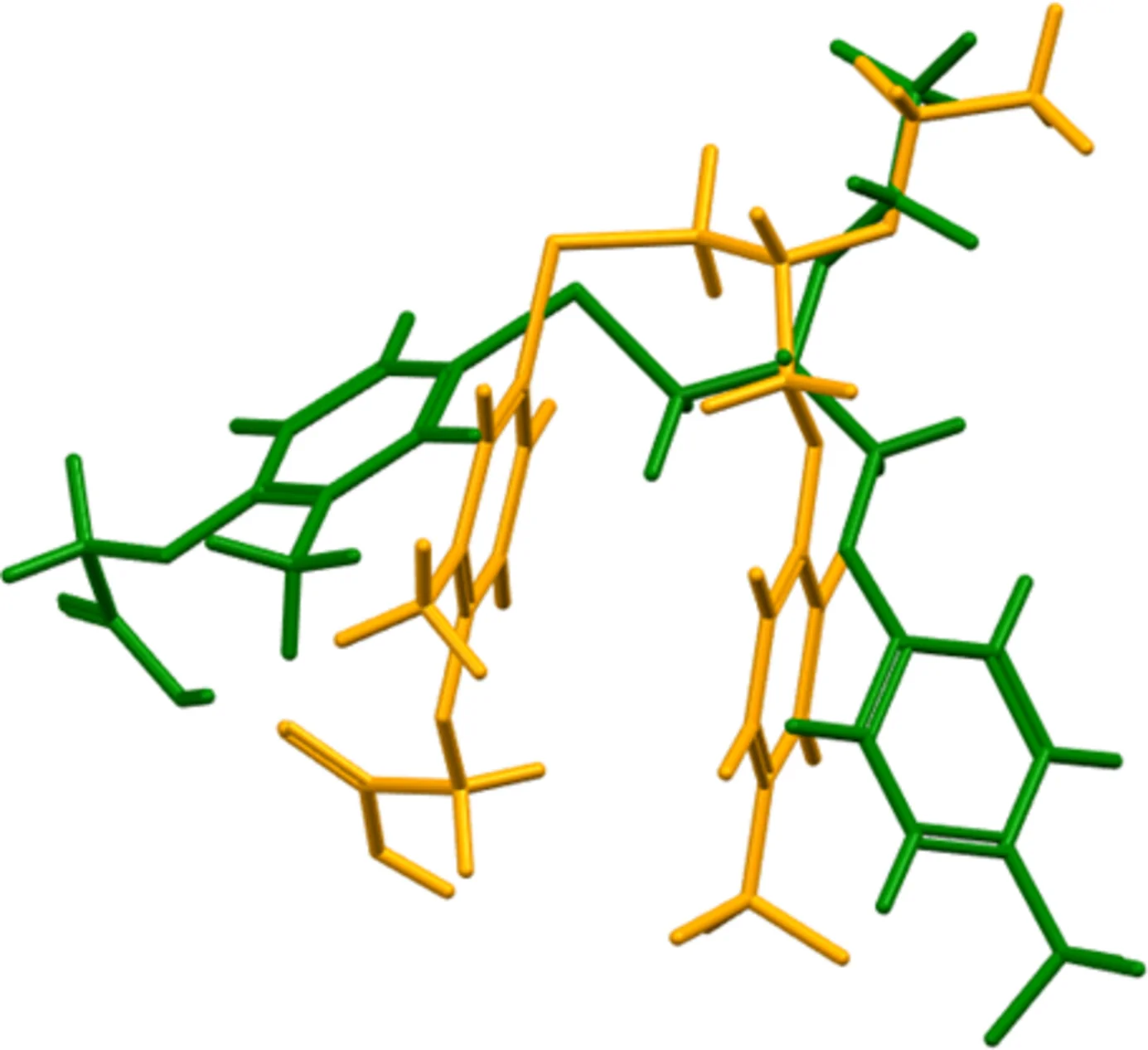
Comparison of the mol­ecular structure of seladelpar determined here (orange) with that in PDB entry 8HUP (green). The root-mean-square difference is 2.801 Å.

Quantum chemical geometry optimization of the isolated seladelpar mol­ecule (DFT/B3LYP/6-31G*/water) using *Spartan ’24* (Wavefunction, 2025 ▸) indicated that the observed conformation is 3.6 kcal mol^−1^ higher in energy than a local minimum, which has a similar overall shape (r.m.s. difference = 0.56 Å) but differences at the periphery of the mol­ecule. The global minimum-energy conformation (MMFF force field) is 8.3 kcal mol^−1^ lower in energy, with a similar overall shape (r.m.s. difference = 1.76 Å) but with differences in the orientations of the carb­oxy­lic acid and tri­fluoro­methyl groups. The conformation of seladelpar in the PDB entry 8HUP is very different from that observed here (Fig. 4 ▸; r.m.s. difference = 2.80 Å) and 1.4 kcal mol^−1^ lower in energy. Apparently, the mol­ecule is flexible, and inter­molecular inter­actions are important in determining the observed conformation.

## 3. Supra­molecular features

**Figure 5:**
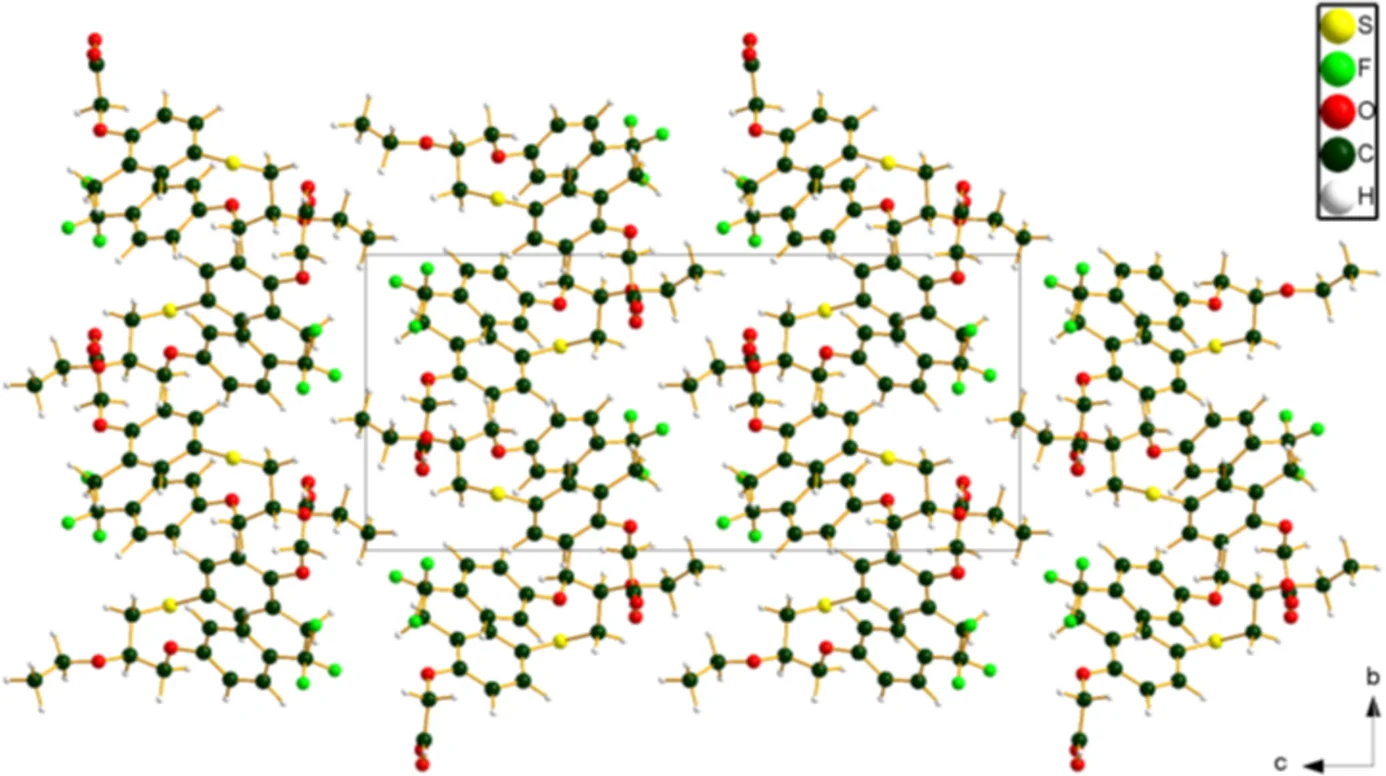
Crystal structure of seladelpar, viewed down the *a*-axis direction.

The crystal structure (Fig. 5 ▸) is characterized by layers lying parallel to the *ab* plane. Parallel stacking of rings is apparent when viewed in different directions. The *Mercury* aromatics analyser indicates two strong (*d* = 5.09 Å) inter­actions between phenyl rings, three moderate (*d* = 5.89, 5.89, and 6.32 Å), and some weaker inter­actions (*d* > 8.7 Å). Analysis of the contributions to the total crystal energy of the structure using the Forcite module of *Materials Studio* (Dassault Systèmes, 2025 ▸) indicated that the intra­molecular energy is dominated by angle distortion terms, and that bond and torsion terms are also significant. The inter­molecular energy is dominated by van der Waals attractions, which in this force field-based analysis include hydrogen bonds. The hydrogen bonds are better discussed using the results of the DFT calculation.

There is one classical O—H⋯O hydrogen bond in the structure (Table 2 ▸). The energy of this bond (14.7 kcal mol^−1^) was calculated using the correlation of Rammohan & Kaduk (2018 ▸). This links the carb­oxy­lic acid and the ether O5 atom into chains propagating along the *b*-axis direction, with graph-set motif (Etter, 1990 ▸; Bernstein *et al.*, 1995 ▸; Motherwell *et al.*, 2000 ▸) *C*(13). A less-traditional (and from the overlap population, surprisingly strong) C—H⋯S hydrogen bond links an aromatic ring and the S atom, and reinforces the chains along the *b*-axis.

**Figure 6:**
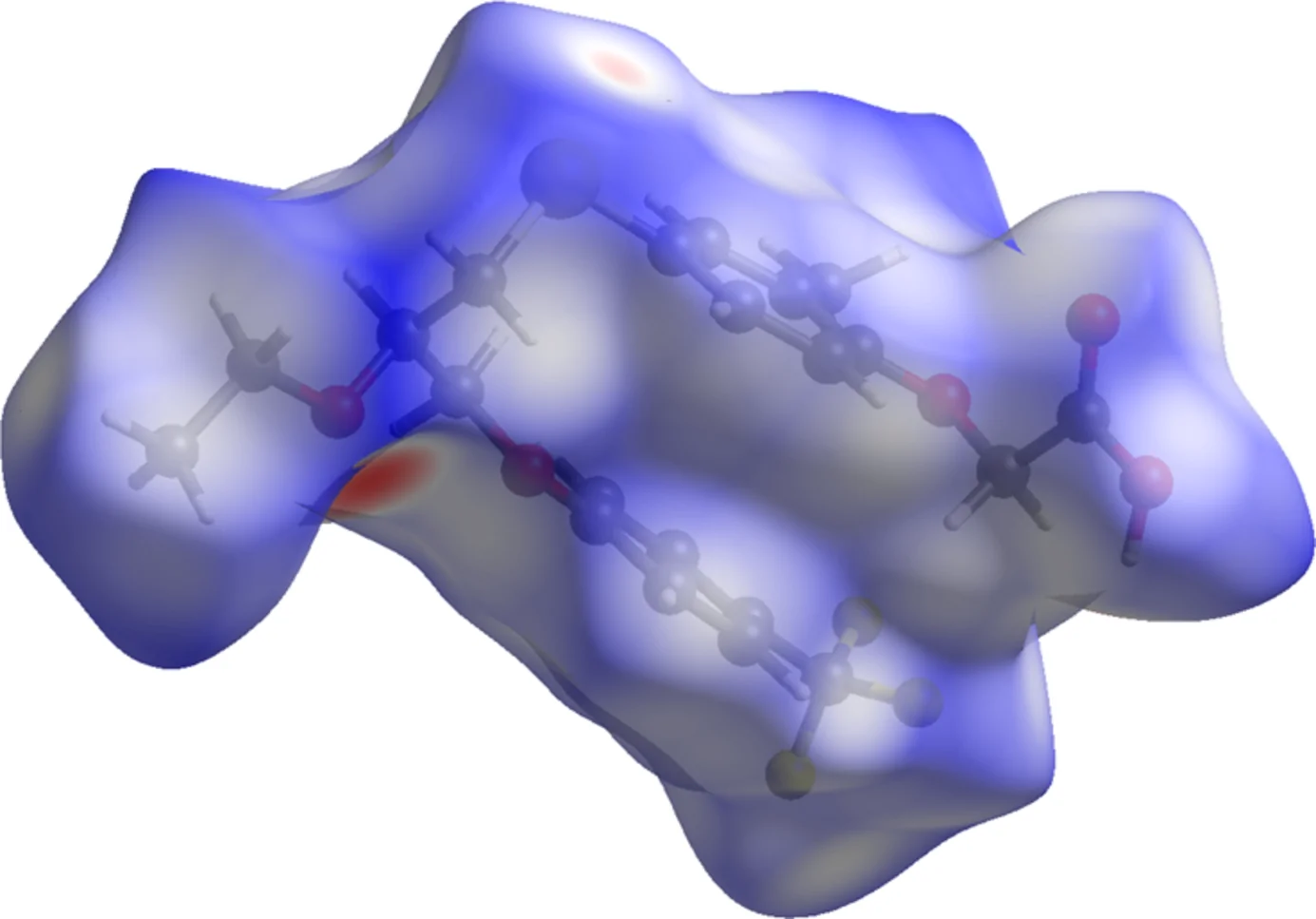
The Hirshfeld surface of seladelpar. Inter­molecular contacts longer than the sums of the van der Waals radii are colored blue, and contacts shorter than the sums of the radii are colored red. Contacts approximately equal to the sums of radii are white.

The volume enclosed by the Hirshfeld surface of seladelpar (Fig. 6 ▸; Hirshfeld, 1977 ▸; Spackman *et al.*, 2021 ▸) is 546.7 Å^3^ or 98.6% of 1/4 of the unit cell volume. The packing density is thus typical. The close contacts (red in Fig. 6 ▸) involve the hydrogen bonds. The volume per non-hydrogen atom is normal, at 18.5 Å^3^.

The Bravais–Friedel–Donnay–Harker (Bravais, 1866 ▸; Friedel, 1907 ▸; Donnay & Harker, 1937 ▸) algorithm suggests that we might expect lozenge morphology for seladelpar, with {001} as the major faces. A second-order spherical harmonic model for preferred orientation was included. The texture index was 1.006, indicating that the preferred orientation was negligible in this rotated capillary specimen.

## 4. Database survey

Powder diffraction data for anhydrous and dihydrate lysine salts of seladelpar are reported in US Patent US 7,709,682 B2 (Abdel-Magid *et al.*, 2010 ▸; Janssen Pharmaceutical). We are unaware of any published powder diffraction data for seladelpar itself. The structures of several proteins complexed with seladelpar, have been determined (Kamata *et al.*, 2023 ▸; PDB entries 8HUN, 8HUO, and 8HUP). A reduced cell search in the Cambridge Structural Database (Groom *et al.*, 2016 ▸), with the chemistry C, H, F, O and S only, yielded no hits.

## 5. Synthesis and crystallization

Seladelpar is a commercial reagent, purchased from TargetMol (Batch #225397), and was used as-received.

## 6. Refinement

Crystal data, data collection and structure refinement details are summarized in Table 3 ▸. The white powder was packed into a 1.5 mm diameter Kapton capillary, and rotated during the measurement at 50 Hz. The powder pattern was measured at 295 K at beam line 11-BM (Lee *et al.*, 2008 ▸; Wang *et al.*, 2008 ▸; Antao *et al.*, 2008 ▸) of the Advanced Photon Source at Argonne National Laboratory using a wavelength of 0.4687342 Å from 0.5–50° 2θ with a step size of 0.001° and a counting time of 0.1 sec step^−1^. The high-resolution powder diffraction data were collected using twelve silicon crystal analyzers that allow for high angular resolution, high precision, and accurate peak positions. A mixture of silicon (NIST SRM 640c) and alumina (NIST SRM 676a) standards (ratio Al_2_O_3_:Si = 2:1 by weight) was used to calibrate the instrument and refine the monochromatic wavelength used in the experiment.

The pattern was indexed on a primitive ortho­rhom­bic unit cell with *a* = 8.87586, *b* = 10.62254, *c* = 23.54278 Å, *V* = 2219.7 Å^3^, and *Z* = 4 using *N-TREOR* as incorporated into *EXPO2014* (Altomare *et al.*, 2013 ▸). The suggested space group was *P*2_1_2_1_2_1_, which was confirmed by the successful solution and refinement of the structure. This cell does not account for a few weak peaks, so the sample contains at least one crystalline impurity.

The mol­ecular structure of seladelpar was downloaded from PubChem (Kim *et al.*, 2023 ▸) as Conformer3D_COMPOUND_CID_11236126.sdf. It was converted to a *.mol2 file using *Mercury* (Macrae *et al.*, 2020 ▸), and to a Fenske–Hall *Z*-matrix using *OpenBabel* (O’Boyle *et al.*, 2011 ▸). The structure was solved using parallel tempering techniques as implemented in *FOX* (Favre-Nicolin & Černý, 2002 ▸).

Rietveld refinement was carried out using *GSAS-II* (Toby & Von Dreele, 2013 ▸). Only the 1.5–19.0° portion of the pattern was included in the refinements (*d*_min_ = 1.420 Å). All non-H bond distances and angles were subjected to restraints, based on a *Mercury*/Mogul Geometry Check (Sykes *et al.*, 2011 ▸; Bruno *et al.*, 2004 ▸). The Mogul average and standard deviation for each qu­antity were used as the restraint parameters. The aromatic rings were restrained to be planar. The restraints contributed 7.3% to the overall *χ^2^*. The hydrogen atoms were included in calculated positions, which were recalculated during the refinement using Mercury. The *U*_iso_ values of the non-H atoms were grouped by chemical similarity. The *U*_iso_ of the H atoms were fixed at 1.2× the *U*_iso_ of the heavy atom to which they are attached. The peak profiles were described using the generalized microstrain model (Stephens, 1999 ▸). The background was modeled using a six-term shifted Chebyshev polynomial, with a peak at 6.08° to model the scattering from the Kapton capillary and any amorphous component of the sample.

**Figure 7:**
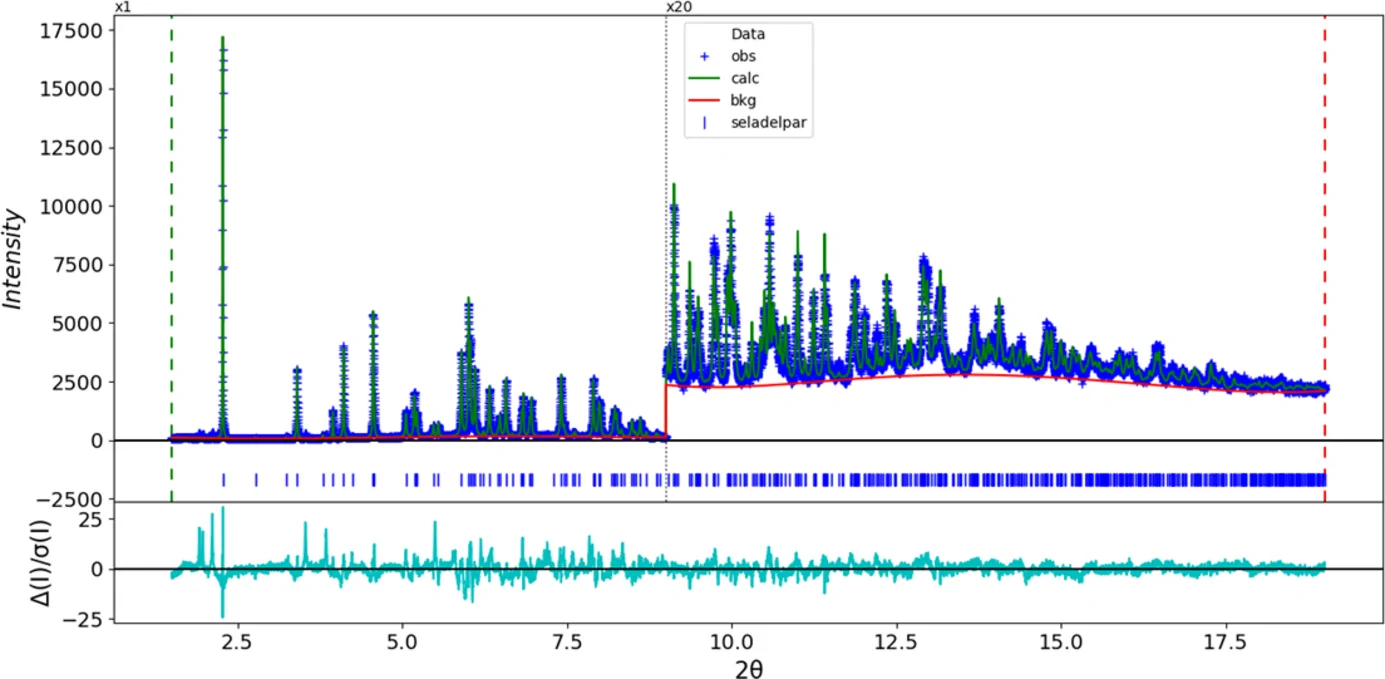
The Rietveld plot for seladelpar. The blue crosses represent the observed data points, and the green line is the calculated pattern. The cyan curve is the normalized error plot, and the red line is the background curve. The blue tick marks indicate the peak positions. The vertical scale has been multiplied by a factor of 20× for 2θ > 9.0°.

The final refinement of 114 variables using 17,501 observations and 75 restraints yielded the residuals *R*_wp_ = 0.0914 and GOF = 3.34. The largest peak (1.21 Å from F4) and hole (1.95 Å from S1) in the difference-Fourier map are 0.23 (4) and −0.20 (4) *e* Å^−3^, respectively. The final Rietveld plot is shown in Fig. 7 ▸. The largest features in the normalized error plot are at the impurity peaks and in the shape of the lowest-angle 002 peak.

The crystal structure of seladelpar was optimized (fixed experimental unit cell) with density functional theory techniques using *VASP* (Kresse & Furthmüller, 1996 ▸) through the *MedeA* graphical inter­face (Materials Design, 2024 ▸). The calculation was carried out on 32 cores of a 144-core (768 Gb memory) HPE Superdome Flex 280 Linux server at North Central College. The calculation used the GGA-PBE functional, a plane wave cutoff energy of 400.0 eV, and a *k*-point spacing of 0.5 Å^−1^ leading to a 2 × 2 × 1 mesh, and took ∼11.9 h. Single-point density functional theory calculations (fixed experimental cell) and population analysis were carried out using *CRYSTAL23* (Erba *et al.*, 2023 ▸). (fixed experimental cell) and population analysis were carried out using *CRYSTAL17* (Dovesi *et al.*, 2018 ▸). The basis sets for the H, C, and O atoms in the calculation were those of Gatti *et al.* (1994 ▸), and those for F and S were from Peintinger *et al.* (2013 ▸). The calculations were run on a 3.5 GHz PC using 8 *k*-points and the B3LYP functional, and took ∼3.2 h. The powder pattern has been submitted to ICDD for inclusion in the Powder Diffraction File (PDF).

## Supplementary Material

Crystal structure: contains datablock(s) seladelpar, seladelpar_VASP. DOI: 10.1107/S2056989026007875/hb8227sup1.cif

CCDC references: 2577790, 2577791

Additional supporting information:  crystallographic information; 3D view; checkCIF report

## Figures and Tables

**Table 1:** Selected torsion angles (°)

C13—S1—C11—C10	−89.2	C11—C10—O5—C14	75.6
S1—C11—C10—C12	60.4	C10—O5—C14—C21	162.4
O5—C10—C11—S1	−177.7	C10—C12—O6—C15	−169.8
C17—C13—S1—C11	125.4	O5—C10—C12—O6	−64.9
C18—C13—S1—C11	−58.4	C19—O7—C29—C30	−82.2
C11—C10—C12—O6	58.8		

**Table 2:** Hydrogen-bond geometry (Å, °)

*D*—H⋯*A*	*D*—H	H⋯*A*	*D*⋯*A*	*D*—H⋯*A*
O8—H1⋯O5^i^	1.01	1.72	2.725	175
C23—H16⋯S1^ii^	1.09	2.72	3.785	166

Symmetry codes: (i) 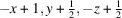; (ii) 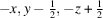.

**Table 3:** Experimental details

Crystal data	
Chemical formula	C_21_H_23_F_3_O_5_S
*M*_r_	444.46
Crystal system, space group	Orthorhombic, *P*2_1_2_1_2_1_
Temperature (K)	295
*a*, *b*, *c* (Å)	8.85689 (7), 10.62563 (8), 23.57851 (14)
*V* (Å^3^)	2218.98 (2)
*Z*	4
Radiation type	Synchrotron, λ = 0.46873 Å
μ (mm^−1^)	0.02
Specimen shape, size (mm)	Cylinder, 2.0 × 1.5

Data collection	
Diffractometer	11-BM, APS
Specimen mounting	Kapton capillary
Data collection mode	Transmission
Scan method	Step
2θ values (°)	2θ_min_ = 0.510, 2θ_max_ = 49.995, 2θ_step_ = 0.001

Refinement	
*R* factors and goodness of fit	*R*_p_ = 0.066, *R*_wp_ = 0.088, *R*_exp_ = 0.027, *R*(*F*^2^) = 0.06486, χ^2^ = 11.169
No. of parameters	114
No. of restraints	75
(Δ/σ)_max_	11.051

Computer programs: *GSAS-II* (Toby & Von Dreele, 2013 ▸).

## supplementary crystallographic information

### 2-(4-{(2R)-2-Ethoxy-3-[4-(trifluoromethyl)phenoxy]propylsulfanyl}-2-methylphenoxy)acetic acid (seladelpar) Crystal data

**Table:**

C_21_H_23_F_3_O_5_S	*Z* = 4
*M**_r_* = 444.46	*D*_x_ = 1.330 Mg m^−^^3^
Orthorhombic, *P*2_1_2_1_2_1_	Synchrotron radiation, λ = 0.46873 Å
*a* = 8.85689 (7) Å	µ = 0.02 mm^−^^1^
*b* = 10.62563 (8) Å	*T* = 295 K
*c* = 23.57851 (14) Å	cylinder, 2.0 × 1.5 mm
*V* = 2218.98 (2) Å^3^	

### 2-(4-{(2R)-2-Ethoxy-3-[4-(trifluoromethyl)phenoxy]propylsulfanyl}-2-methylphenoxy)acetic acid (seladelpar) Data collection

**Table:**

11-BM, APS diffractometer	Scan method: step
Specimen mounting: Kapton capillary	2θ_min_ = 0.510°, 2θ_max_ = 49.995°, 2θ_step_ = 0.001°
Data collection mode: transmission	

### 2-(4-{(2R)-2-Ethoxy-3-[4-(trifluoromethyl)phenoxy]propylsulfanyl}-2-methylphenoxy)acetic acid (seladelpar) Refinement

**Table:**

Least-squares matrix: full	114 parameters
*R*_p_ = 0.066	75 restraints
*R*_wp_ = 0.088	25 constraints
*R*_exp_ = 0.027	Weighting scheme based on measured s.u.'s
*R*(*F*^2^) = 0.06486	(Δ/σ)_max_ = 11.051
49486 data points	Background function: Background function: "chebyschev-1" function with 6 terms: 83.2(15), 38.6(18), 1.6(7), -42.6(15), 20.8(9), 3.7(4), Background peak parameters: pos, int, sig, gam: 6.080(24), 7.1(3)e4, 3.46(12)e4, 0.100,
Profile function: Finger-Cox-Jephcoat function parameters U, V, W, X, Y, SH/L: peak variance(Gauss) = Utan(Th)^2^+Vtan(Th)+W: peak HW(Lorentz) = X/cos(Th)+Ytan(Th); SH/L = S/L+H/L U, V, W in (centideg)^2^, X & Y in centideg 1.163, -0.126, 0.063, 0.000, 0.000, 0.002,	Preferred orientation correction: Simple spherical harmonic correction Order = 2 Coefficients: 0:0:C(2,0) = -0.1570; 0:0:C(2,2) = 0.0680

### 2-(4-{(2R)-2-Ethoxy-3-[4-(trifluoromethyl)phenoxy]propylsulfanyl}-2-methylphenoxy)acetic acid (seladelpar) Fractional atomic coordinates and isotropic or equivalent isotropic displacement parameters (Å^2^)

**Table:**

	*x*	*y*	*z*	*U*_iso_*/*U*_eq_	
S1	−0.0693 (5)	0.8031 (4)	0.2987 (2)	0.090 (2)*	
F2	0.7564 (9)	0.5457 (7)	0.0969 (4)	0.110 (2)*	
F3	0.7032 (8)	0.7385 (6)	0.0780 (3)	0.110 (2)*	
F4	0.5772 (9)	0.5866 (7)	0.0556 (3)	0.110 (2)*	
O5	0.2116 (8)	0.6317 (8)	0.4072 (4)	0.074 (2)*	
O6	0.3274 (10)	0.6649 (8)	0.2992 (4)	0.074 (2)*	
O7	0.3205 (9)	0.9186 (8)	0.0999 (3)	0.091 (2)*	
O8	0.4964 (8)	1.2284 (8)	0.0955 (5)	0.091 (2)*	
O9	0.2451 (9)	1.1752 (7)	0.0908 (5)	0.091 (2)*	
C10	0.1126 (10)	0.6346 (9)	0.3592 (4)	0.074 (2)*	
C11	0.0640 (11)	0.7684 (10)	0.3557 (4)	0.074 (2)*	
C12	0.1936 (12)	0.5916 (10)	0.3060 (4)	0.074 (2)*	
C13	0.0490 (9)	0.8496 (9)	0.2404 (3)	0.038 (3)*	
C14	0.1323 (11)	0.6125 (11)	0.4623 (5)	0.074 (2)*	
C15	0.4075 (14)	0.6504 (12)	0.2525 (4)	0.110 (2)*	
C16	0.1267 (10)	0.8081 (8)	0.1427 (3)	0.038 (3)*	
C17	0.0330 (9)	0.7833 (8)	0.1890 (4)	0.038 (3)*	
C18	0.1583 (11)	0.9472 (8)	0.2440 (3)	0.038 (3)*	
C19	0.2459 (13)	0.8937 (11)	0.1496 (3)	0.038 (3)*	
C20	0.2492 (11)	0.9734 (9)	0.1961 (4)	0.038 (3)*	
C21	0.2342 (15)	0.5533 (13)	0.4997 (5)	0.074 (2)*	
C22	0.5226 (13)	0.7313 (10)	0.2432 (4)	0.110 (2)*	
C23	0.3515 (14)	0.5777 (12)	0.2093 (4)	0.110 (2)*	
C24	0.5616 (11)	0.6439 (11)	0.1505 (4)	0.110 (2)*	
C25	0.1227 (10)	0.7196 (8)	0.0935 (4)	0.038 (3)*	
C26	0.5977 (12)	0.7303 (11)	0.1910 (4)	0.110 (2)*	
C27	0.4405 (10)	0.5645 (11)	0.1606 (3)	0.110 (2)*	
C28	0.6537 (8)	0.6326 (6)	0.0973 (3)	0.110 (2)*	
C29	0.4303 (10)	1.0128 (8)	0.0970 (5)	0.091 (2)*	
C30	0.3809 (9)	1.1448 (7)	0.0919 (8)	0.091 (2)*	
H1	0.59404	1.18258	0.09406	0.1095*	
H2	0.01509	0.57462	0.36702	0.0882*	
H3	0.16412	0.82616	0.34929	0.0882*	
H4	0.01101	0.79351	0.39581	0.0882*	
H5	0.22332	0.49253	0.30988	0.0882*	
H6	0.12036	0.60460	0.26937	0.0882*	
H7	0.09737	0.70291	0.47965	0.0882*	
H8	0.03346	0.55313	0.45607	0.0882*	
H9	−0.05405	0.71139	0.18531	0.0453*	
H10	0.17186	1.00095	0.28299	0.0453*	
H11	0.32173	1.05616	0.19577	0.0453*	
H12	0.33292	0.61300	0.50555	0.0882*	
H13	0.26901	0.46322	0.48197	0.0882*	
H14	0.17938	0.53796	0.54046	0.0882*	
H15	0.55678	0.79704	0.27622	0.1322*	
H16	0.24187	0.53194	0.21275	0.1322*	
H17	0.02154	0.66162	0.09621	0.0453*	
H18	0.22226	0.65950	0.09449	0.0453*	
H19	0.12138	0.77315	0.05412	0.0453*	
H20	0.68590	0.79927	0.18265	0.1322*	
H21	0.41411	0.49062	0.13016	0.1322*	
H22	0.49806	1.00599	0.13542	0.1095*	
H23	0.50063	0.99235	0.06025	0.1095*	

### 2-(4-{(2R)-2-Ethoxy-3-[4-(trifluoromethyl)phenoxy]propylsulfanyl}-2-methylphenoxy)acetic acid (seladelpar) Geometric parameters (Å, º)

**Table:**

S1—C11	1.826 (6)	C21—C14	1.410 (14)
S1—C13	1.798 (5)	C21—H12	1.089 (13)
F2—F3	2.149 (9)	C21—H13	1.088 (13)
F2—F4	1.912 (9)	C21—H14	1.090 (13)
F2—C28	1.296 (8)	C22—C15	1.352 (6)
F3—F2	2.149 (9)	C22—C26	1.399 (6)
F3—F4	2.032 (9)	C22—H15	1.089 (6)
F3—C28	1.291 (8)	C23—C15	1.371 (7)
F4—F2	1.912 (9)	C23—C27	1.399 (7)
F4—F3	2.032 (9)	C23—H16	1.089 (8)
F4—C28	1.290 (8)	C24—C26	1.362 (5)
O5—C10	1.431 (7)	C24—C27	1.385 (5)
O5—C14	1.491 (9)	C24—C28	1.502 (5)
O6—C12	1.428 (8)	C25—C16	1.494 (6)
O6—C15	1.319 (6)	C25—H17	1.089 (9)
O7—C19	1.370 (6)	C25—H18	1.089 (9)
O7—C29	1.397 (6)	C25—H19	1.089 (10)
O8—C30	1.357 (7)	C26—C22	1.399 (6)
O8—H1	0.993 (7)	C26—C24	1.362 (5)
O9—C30	1.246 (8)	C26—H20	1.089 (7)
C10—O5	1.431 (7)	C27—C23	1.399 (7)
C10—C11	1.488 (7)	C27—C24	1.385 (5)
C10—C12	1.516 (8)	C27—H21	1.090 (7)
C10—H2	1.089 (9)	C28—F2	1.296 (8)
C11—S1	1.826 (6)	C28—F3	1.291 (8)
C11—C10	1.488 (7)	C28—F4	1.290 (8)
C11—H3	1.089 (11)	C28—C24	1.502 (5)
C11—H4	1.088 (10)	C29—O7	1.397 (6)
C12—O6	1.428 (8)	C29—C30	1.474 (7)
C12—C10	1.516 (8)	C29—H22	1.089 (12)
C12—H5	1.089 (11)	C29—H23	1.089 (10)
C12—H6	1.089 (11)	C30—O8	1.357 (7)
C13—S1	1.798 (5)	C30—O9	1.246 (8)
C13—C17	1.409 (5)	C30—C29	1.474 (7)
C13—C18	1.421 (6)	H1—O8	0.993 (7)
C14—O5	1.491 (9)	H2—C10	1.089 (9)
C14—C21	1.410 (14)	H3—C11	1.089 (11)
C14—H7	1.089 (11)	H4—C11	1.088 (10)
C14—H8	1.089 (11)	H5—C12	1.089 (11)
C15—O6	1.319 (6)	H6—C12	1.089 (11)
C15—C22	1.352 (6)	H7—C14	1.089 (11)
C15—C23	1.371 (7)	H8—C14	1.089 (11)
C16—C17	1.396 (4)	H9—C17	1.089 (6)
C16—C19	1.403 (6)	H10—C18	1.089 (7)
C16—C25	1.494 (6)	H11—C20	1.089 (7)
C17—C13	1.409 (5)	H12—C21	1.089 (13)
C17—C16	1.396 (4)	H13—C21	1.088 (13)
C17—H9	1.089 (6)	H14—C21	1.090 (13)
C18—C13	1.421 (6)	H15—C22	1.089 (6)
C18—C20	1.414 (6)	H16—C23	1.089 (8)
C18—H10	1.089 (7)	H17—C25	1.089 (9)
C19—O7	1.370 (6)	H18—C25	1.089 (9)
C19—C16	1.403 (6)	H19—C25	1.089 (10)
C19—C20	1.387 (6)	H20—C26	1.089 (7)
C20—C18	1.414 (6)	H21—C27	1.090 (7)
C20—C19	1.387 (6)	H22—C29	1.089 (12)
C20—H11	1.089 (7)	H23—C29	1.089 (10)
			
C11—S1—C13	104.0 (4)	C16—C19—C20	120.2 (4)
C10—O5—C14	113.8 (5)	C18—C20—C19	120.0 (4)
C12—O6—C15	118.4 (6)	C18—C20—H11	120.1 (7)
C19—O7—C29	121.1 (6)	C19—C20—H11	119.9 (7)
C30—O8—H1	109.5 (7)	C14—C21—H12	109.5 (12)
O5—C10—C11	104.0 (6)	C14—C21—H13	109.5 (11)
O5—C10—C12	110.9 (7)	H12—C21—H13	109.5 (11)
C11—C10—C12	112.3 (7)	C14—C21—H14	109.4 (11)
O5—C10—H2	109.9 (7)	H12—C21—H14	109.4 (11)
C11—C10—H2	109.8 (9)	H13—C21—H14	109.5 (12)
C12—C10—H2	109.8 (8)	C15—C22—C26	119.7 (3)
S1—C11—C10	114.9 (6)	C15—C22—H15	120.1 (7)
S1—C11—H3	108.1 (6)	C26—C22—H15	120.1 (7)
C10—C11—H3	108.1 (8)	C15—C23—C27	117.5 (5)
S1—C11—H4	108.1 (7)	C15—C23—H16	121.3 (8)
C10—C11—H4	108.1 (8)	C27—C23—H16	121.2 (7)
H3—C11—H4	109.5 (8)	C26—C24—C27	118.1 (3)
O6—C12—C10	108.8 (6)	C26—C24—C28	120.8 (4)
O6—C12—H5	109.6 (10)	C27—C24—C28	121.1 (4)
C10—C12—H5	109.7 (9)	C16—C25—H17	109.3 (6)
O6—C12—H6	109.6 (10)	C16—C25—H18	109.4 (9)
C10—C12—H6	109.7 (8)	H17—C25—H18	109.5 (8)
H5—C12—H6	109.5 (7)	C16—C25—H19	109.5 (8)
S1—C13—C17	117.5 (5)	H17—C25—H19	109.7 (8)
S1—C13—C18	123.5 (6)	H18—C25—H19	109.5 (7)
C17—C13—C18	119.0 (3)	C22—C26—C24	120.7 (4)
O5—C14—C21	107.7 (8)	C22—C26—H20	119.7 (6)
O5—C14—H7	109.9 (11)	C24—C26—H20	119.7 (7)
C21—C14—H7	109.9 (10)	C23—C27—C24	121.1 (4)
O5—C14—H8	109.9 (8)	C23—C27—H21	119.5 (6)
C21—C14—H8	109.9 (12)	C24—C27—H21	119.4 (6)
H7—C14—H8	109.5 (9)	F2—C28—F3	112.4 (6)
O6—C15—C22	117.8 (6)	F2—C28—F4	95.3 (6)
O6—C15—C23	119.5 (7)	F3—C28—F4	103.8 (6)
C22—C15—C23	120.7 (4)	F2—C28—C24	116.4 (6)
C17—C16—C19	118.6 (3)	F3—C28—C24	114.2 (5)
C17—C16—C25	118.3 (4)	F4—C28—C24	112.4 (5)
C19—C16—C25	121.1 (4)	O7—C29—C30	118.6 (7)
C13—C17—C16	121.2 (3)	O7—C29—H22	107.1 (8)
C13—C17—H9	119.3 (6)	C30—C29—H22	107.1 (11)
C16—C17—H9	119.4 (6)	O7—C29—H23	107.1 (8)
C13—C18—C20	118.9 (4)	C30—C29—H23	107.2 (11)
C13—C18—H10	120.6 (7)	H22—C29—H23	109.5 (8)
C20—C18—H10	120.5 (8)	O8—C30—O9	124.0 (6)
O7—C19—C16	112.9 (5)	O8—C30—C29	113.2 (6)
O7—C19—C20	123.2 (6)	O9—C30—C29	122.3 (6)

### (seladelpar_VASP) Crystal data

**Table:**

C_21_H_23_F_3_O_5_S	*b* = 10.62563 Å
*M**_r_* = 444.46	*c* = 23.57851 Å
Orthorhombic, *P*2_1_2_1_2_1_	*V* = 2218.97 Å^3^
*a* = 8.85689 Å	*Z* = 4

### (seladelpar_VASP) Data collection

**Table:**

*h* = →	*l* = →
*k* = →	

### (seladelpar_VASP) Fractional atomic coordinates and isotropic or equivalent isotropic displacement parameters (Å^2^)

**Table:**

	*x*	*y*	*z*	*B*_iso_*/*B*_eq_	
S1	−0.06226	0.81067	0.29800		
F2	0.77456	0.54746	0.09473		
F3	0.71686	0.74416	0.07619		
F4	0.56934	0.59128	0.04659		
O5	0.21714	0.62095	0.40710		
O6	0.32888	0.66722	0.29666		
O7	0.31495	0.92206	0.09562		
O8	0.50360	1.22795	0.08729		
O9	0.25766	1.17937	0.08604		
C10	0.11756	0.64131	0.35934		
C11	0.06945	0.77962	0.35565		
C12	0.19659	0.59245	0.30683		
C13	0.05734	0.84570	0.23965		
C14	0.14147	0.61905	0.46174		
C15	0.40328	0.65185	0.24652		
C16	0.12593	0.80438	0.14067		
C17	0.04109	0.77718	0.18907		
C18	0.16162	0.94442	0.24135		
C19	0.23206	0.90300	0.14406		
C20	0.24941	0.97255	0.19398		
C21	0.24516	0.55791	0.50448		
C22	0.52414	0.73468	0.23699		
C23	0.36658	0.56059	0.20585		
C24	0.56755	0.63829	0.14545		
C25	0.11139	0.72919	0.08716		
C26	0.60502	0.72797	0.18674		
C27	0.44978	0.55388	0.15579		
C28	0.65496	0.63091	0.09138		
C29	0.43670	1.00800	0.09718		
C30	0.38886	1.14525	0.08996		
H1	0.60569	1.18687	0.09182		
H2	0.01570	0.58277	0.36528		
H3	0.16772	0.84182	0.35156		
H4	0.00738	0.80551	0.39423		
H5	0.22856	0.49313	0.31316		
H6	0.11867	0.59834	0.27056		
H7	0.11314	0.71583	0.47476		
H8	0.03500	0.56616	0.45721		
H9	−0.03852	0.69872	0.18759		
H10	0.17493	1.00072	0.27971		
H11	0.32779	1.05128	0.19648		
H12	0.35153	0.61041	0.50867		
H13	0.26913	0.45973	0.49293		
H14	0.19102	0.55929	0.54627		
H15	0.55108	0.80474	0.26919		
H16	0.27245	0.49580	0.21180		
H17	0.02872	0.65325	0.09216		
H18	0.22091	0.68798	0.07532		
H19	0.07661	0.78864	0.05126		
H20	0.69791	0.79322	0.17921		
H21	0.42195	0.48166	0.12463		
H22	0.50381	0.99822	0.13638		
H23	0.51065	0.98199	0.06162		

### (seladelpar_VASP) Torsion angles (º)

**Table:**

C13—S1—C11—C10	−89.2	C11—C10—O5—C14	75.6
S1—C11—C10—C12	60.4	C10—O5—C14—C21	162.4
O5—C10—C11—S1	−177.7	C10—C12—O6—C15	−169.8
C17—C13—S1—C11	125.4	O5—C10—C12—O6	−64.9
C18—C13—S1—C11	−58.4	C19—O7—C29—C30	−82.2
C11—C10—C12—O6	58.8		

### (seladelpar_VASP) Hydrogen-bond geometry (Å, º)

**Table:**

*D*—H···*A*	*D*—H	H···*A*	*D*···*A*	*D*—H···*A*
O8—H1···O5^i^	1.01	1.72	2.725	175
C23—H16···S1^ii^	1.09	2.72	3.785	166

Symmetry codes: (i) −*x*+1, *y*+1/2, −*z*+1/2; (ii) −*x*, *y*−1/2, −*z*+1/2.
